# California Wines

**Published:** 1868-12-15

**Authors:** 


### CALIFORNIA WINES.

IN a former number of this JOURNAL we called attention to the Wines introduced in this market by the El Dorado Wine Company. These Wines fully sustain the endorsement we then gave them. The fact of the Company manufacturing their own Wine and Brandy; the reputation of the gentlemen, many of whom are old residents of this city, connected with it, is a sufficient guarantee that their Wines are as represented—*pure* and *unadulterated*; then add to this the rigid tests to which they have been subjected (being of recent introduction here), only confirms our former opinion.

Every one knows the necessity for *Pure Wines* for the sick-room and hospitals. Too great care cannot be observed. We again commend the Wines of this Company, and shall hope that every Drug Store will keep them—that the profession, in prescribing, may always find them readily at hand.

The trade-mark of the Company is peculiar (bear and face), and can not well be mistaken. The office of the Company is at 122 South Clark Street.

